# A meta-analysis of colchicine in prevention of atrial fibrillation following cardiothoracic surgery or cardiac intervention

**DOI:** 10.1186/s13019-022-01958-9

**Published:** 2022-09-01

**Authors:** Hong Zhao, Yueming Chen, Min Mao, Jun Yang, Jing Chang

**Affiliations:** 1 Department of Cardiology, First Affiliated Hospital of Chongqing Medical University, Yuzhong District, Chongqing, 40000 China; 2 Department of General Practice, The First Affiliated Hospital of Chongqing Medical University, Yuzhong District, Chongqing, 40000 China

**Keywords:** Colchicine, Atrial fibrillation, POAF, Cardiothoracic surgery, Cardiac intervention

## Abstract

**Background:**

Postoperative atrial fibrillation (POAF) is the most common complication after cardiothoracic surgery or cardiac intervention. Colchicine is an anti-inflammatory agent that was associated with improved cardiovascular outcomes. However, its effect on POAF prevention was inconsistent across studies. Therefore, the aim of this meta-analysis was to evaluate the efficacy of colchicine in prevention of POAF.

**Methods:**

We searched PubMed, Embase, ClinicalTrials.gov, Cochrane Library database and Google Scholar for randomized controlled trials (RCTs), using terms "atrial fibrillation” and “colchicine". The primary end point was the occurrence of clinically diagnosed atrial fibrillation. The relative risk (RR) and 95% confidence interval (CI) were evaluated. Estimates were pooled using DerSimonian-Laird random-effects model. We also performed subgroup analyses based on the duration and dose of colchicine treatment.

**Results:**

A total of 9 RCTs were included in this meta-analysis, enrolling a total of 2031 patients. Colchicine significantly reduces the incidence of POAF (RR 0.62; 95% CI, 0.52–0.74, *P* < 0.001, *I*^*2*^ = 0%). Subgroup analyses indicated that the protective effect of colchicine on POAF was slightly stronger in the long-duration group (RR 0.60; 95% CI, 0.48–0.75, *P* < 0.001, *I*^2^ = 0%) than in the short-duration group (RR 0.65; 95% CI, 0.49–0.86, *P* < 0.001, *I*^2^ = 0%).

**Conclusion:**

Colchicine is effective in preventing the occurrence of POAF. The efficacy of colchicine can be slightly increased over treatment duration, with no obvious adverse reactions.

## Introduction

Post-operative atrial fibrillation (POAF) is a common complication detected following cardiothoracic surgery or cardiac intervention [1]. In this paper, we expand the definition of POAF to include recurrent atrial fibrillation after the cardiac intervention, and cardiac interventions refer primarily to pulmonary vein isolation or ablation. In cardiothoracic surgery, the incidence of POAF has been reported to range between 10 and 50%, with patients undergoing valve surgery being at the highest risk [1–3]. Furthermore, atrial fibrillation (AF) recurrence after the cardiac intervention is expected within the first month, and the inflammatory process may be associated with new-onset AF [4]. POAF is associated with deleterious clinical outcomes, including prolonged hospital stay, expanded treatment costs, and increased morbidity and mortality.

Since inflammatory processes may play an essential role in the pathogenesis of POAF [2], there has been increasing interest in the potential use of anti-inflammatory agents as therapeutics [5]. Colchicine, extracted from the autumn crocus, is an anti-inflammatory agent initially used to treat acute gouty arthritis and subsequently used in Familial Mediterranean Fever [6, 7]. Recent evidence has demonstrated that colchicine reduces adverse events in patients with cardiovascular disease [8, 9]. Some clinical trials and meta-analyses suggested that colchicine has the potential to prevent the occurrence of POAF [5, 10, 11]. However, other studies showed that colchicine was not significantly associated with a lower risk of atrial fibrillation [12]. Besides, previous meta-analyses did not investigate the effect of colchicine dose and duration on the risk of POAF. Therefore, in this study, we performed a systematic review and meta-analysis of randomized clinical trials (RCTs) to obtain estimates of the overall effect of colchicine on POAF prevention. Furthermore, we performed subgroup analyses based on the duration and dose of colchicine treatment.

## Method

This meta-analysis is reported in accordance with the Preferred Reporting Items for Systematic Reviews and Meta-Analysis (PRISMA) Statement and was registered at the International Platform of Registered Systematic Review and Meta-analysis Protocols (registration number: INPLASY202190004) [13, 14]. Moreover, the screening and the review of the full text were done by two authors (Hong Z. and Jing C).

### Search strategy and inclusion criteria

A systematic search for eligible studies was conducted, and relevant articles were retrieved until January 2022 by searching PubMed, Embase, ClinicalTrials.gov, the Cochrane Library, and Google Scholar using the following keywords: “colchicine” AND (“atrial fibrillation” OR “AF”). We also retrieved the citations of the published reviews and meta-analyses to identify additional research.

The inclusion criteria were as follows: (1) Randomized controlled trial (RCT) with the intervention of colchicine or placebo; (2) patients who underwent cardiothoracic surgery or cardiac intervention; (3) the primary endpoint is the occurrence of clinically diagnosed atrial fibrillation. Two authors (Jing C. and Hong Z.) independently reviewed the 149 identified articles. Finally, we included 9 RCTs that evaluated the efficacy of colchicine in preventing atrial fibrillation after cardiothoracic surgery or cardiac intervention [15–23].

### Data extraction

Two reviewers (Jing C. and Hong Z.) separately extracted data from the full-text studies. Any disagreements were resolved by consensus. Data extraction included the name of the first author, year of publication, study design, number of events in the experimental group, total number of events in the experimental group, number of events in the control group, total number of events in the control group, intervention method, intervention time, follow-up time, surgical method, primary endpoint event, and population characteristics (median age and gender).

### Quality assessment

The same two authors (Jing C. and Hong Z.) independently evaluated the methodological quality of the included articles using the Cochrane risk of bias (ROB) tool [24]. The evaluation indicators include sequence generation, allocation concealment, blinding of patients and personnel, blinding of outcome assessment, incomplete outcome data, selective outcome reporting, and bias from other sources. Disagreements were settled by discussion. Each indicator was determined to have a low, high, or unclear risk of bias. The corresponding literature is considered low risk when all the above indicators are low risk. The corresponding literature is classified as high risk as long as there is a high risk. The literature in other situations is classified as unclear risk.

### Statistical analysis

Cochrane collaboration network special software (RevMan 5.0) was used to process data. Heterogeneity was evaluated through Cochran’s Q test, Tau-square, Chi-square, and I-square (*I*^2^) statistics [25]. Statistically, significance was set at a two-tailed *P* < 0.05. Meta-analysis was performed using DerSimonian-Laird random-effect model if the heterogeneity was evident (*I*^2^ ≥ 50%). Otherwise, both the fixed-model and random-effect model were selectable. Relative risk (RR) and 95% confidence interval (CI) were estimated based on the observed proportions of atrial fibrillation in both arms of the study. Results from each included study were combined by fixed-effects inverse-variance-weighted meta-analysis. Subgroup analyses were performed according to the medication duration and surgical approaches. The medication duration subgroup is classified into the long-term group (greater than or equal to one month) and the short-term group (less than one month). The subgroup of surgical approaches is divided into surgery group and intervention group. The relationship between medication duration and adverse drug reactions was also analyzed.

Funnel plots were used to assess potential publication bias and small-study bias. For the sensitivity analysis of the original data, we will exclude each piece of literature one by one and then combine the remaining literature. We compare the result after exclusion with that before exclusion to see if it is statistically different. The sensitivity analysis is used to reflect the stability of the initial results. The statistical procedure will be performed by Stata 14.0 software. We implement the GRADE framework to establish the certainty of the evidence.

## Results

### Characteristics of the included studies

**Fig. 1:**
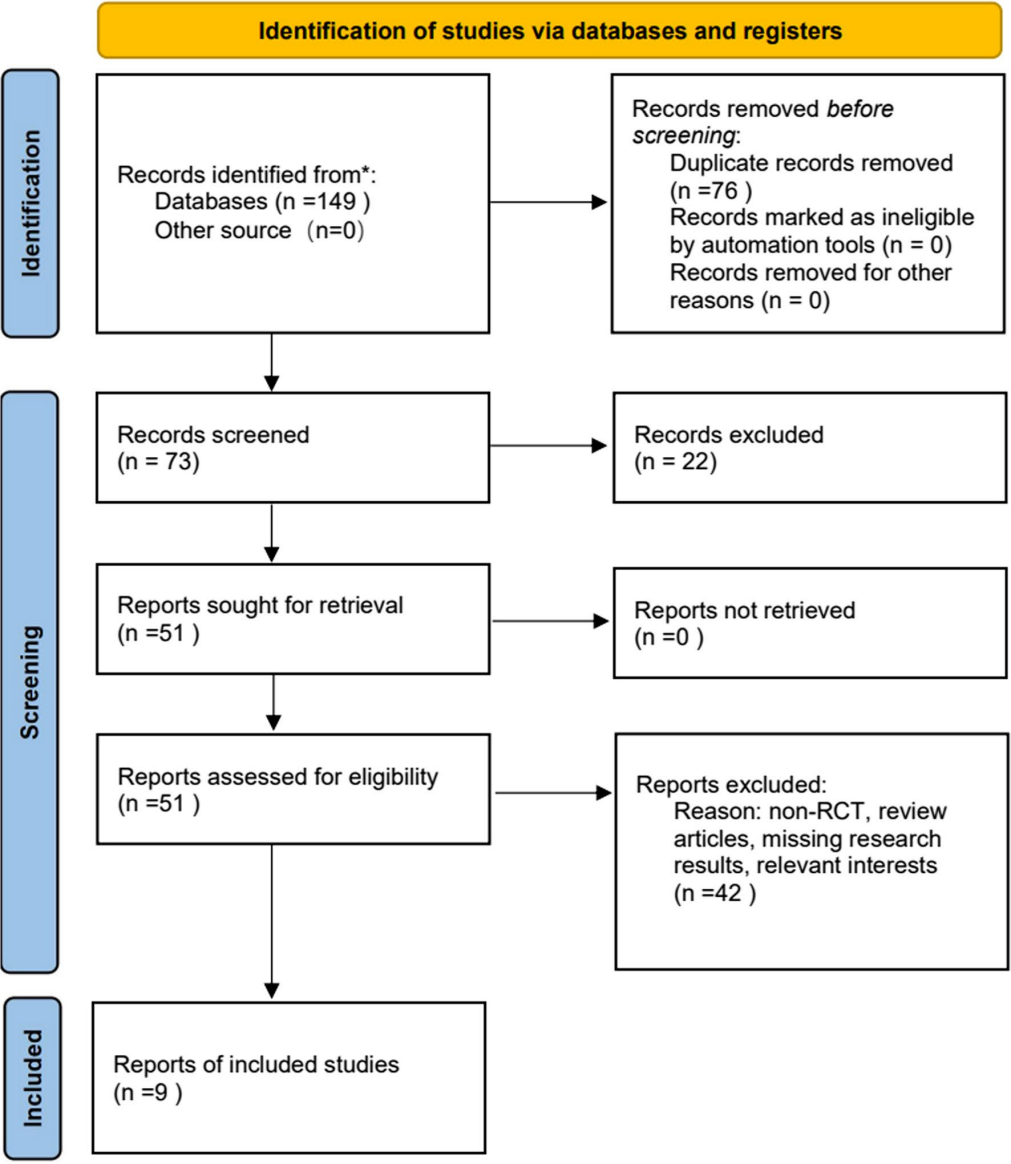
Flow chart of the literature search

One hundred and forty-nine articles were identified, and 9 possibly pertinent articles were chosen after a primary search. Nine studies involving 2031 patients were finally included in our analysis [15–23] (Fig. 1). Seven RCTs were divided into the surgical group (6 RCTs enrolled patients who underwent cardiac surgery [18–23], and 1 RCT enrolled patients who underwent thoracic surgery [17]), while 2 RCTs were divided into intervention group [15, 16]. For medication duration, 5 RCTs were divided into short-term group [17, 20–23], and 4 RCTs were divided into long-term group [15, 16, 18, 19]. The articles described adverse events as gastrointestinal symptoms (including diarrhea and nausea) or directly summarized them as adverse events. The characteristics of the 9 studies and patients are summarized in Table 1.

**Table 1:** Characteristics of RCTs included in the meta-analysis

Study name	Sample size	Intervention	Observation time	Surgical type	Monitoring of AF	Aderverse drug reaction (E/C)	Main terminal point	AF before surgery (E/C)
Bessissow [17]	100	0.6 mg before operation, 0.6 mg bid for 9 day after operation	30 days	Lung resection surgery	(ECG) was per-formed once a day for the first 3 days. Then 30-day follow-up	5/1	POAF	0/0
Deftereos [15]	161	0.5 mg bid for 3 months	3 months	Pulmonary Vein Isolation	Electrogram obtained during patient visits at the arrhythmia clinic	7/1	Recurrence of AF	All/All
Deftereos [16]	206	0.5 mg bid for 3 months	3 months	Pulmonary Vein Isolation	Not report	10/2	Recurrence of AF	All/All
Imazio [19]	336	0.5 mg bid for 1 month	1 month	CABG, aortic surgery, valvular surgery, combined	Continuous ECG monitoring and 12-lead ECG recordings	16/8	Rate of POAF on placebo/colchicine treatment	8/11
Imazio [18]	360	0.5 mg bid for 1 month	3 months	CABG, aortic surgery, valvular surgery, combined	Continuous ECG monitoring and 12-lead ECG recordings	36/21	AF	18/15
Sarzaeem [23]	216	24 h before surgery 2 mg; 0.5 mg bid for 1 week after operation	In hospital	CABG	Continuous ECG monitoring while in ICU, and daily 12 lead ECG while on the regular floor	Not report	AF	Not recorded
Tabbalat [22]	360	0.5 mg bid after operation was taken in hospital until discharge	In hospital	CABG or other open heart surgery	Daily electrocardiograms (ECGs) until discharge	55/14	AF	Not recorded
Tabbalat [21]	152	0.5 mg qd after operation	In hospital	Open heart surgery	Daily electrocardiograms (ECGs) until discharge	2/2	POAF	Not recorded
Zarpelon [20]	140	After operation 0.5 mg bid was taken in hospital until discharge	In hospital	Open heart surgery-Myocardial Revascularization	continuous cardiac monitoring and 12-lead electrocardiogram during ICU stay	19/6	AF	Not recorded

*CABG* coronary artery bypass grafting; *AF* atrial fibrillation; *E/C* experimental group/control group. POAF (in this article) = atrial fibrillation following cardiothoracic surgery or cardiac intervention

Handling of non-English articles, conference literature, and articles with missing information: Firstly, the non-English articles were mainly extracted from their abstract parts,and the translation problems were solved by translation software [23]. Secondly, for handling missing data problems and conference abstracts, the key is whether it affects the conduct of meta-analysis. If critical data are missing, we will exclude the articles. If the non-critical information is incomplete, we define it as high-risk article [20]. The conference literature was not included in this article.

All the 9 RCTs were blinded and placebo-controlled [15–23]. None of the trials was terminated early, even if some participants in individual trials withdrew from the trial due to adverse reactions [15, 16, 18, 19]. Two RCTs did not describe the random sequence generation and allocation concealment in detail and accurately [15, 16]. Four RCTs did not describe whether the result evaluator was blinded [20–23].

### Main outcome

**Fig. 2:**
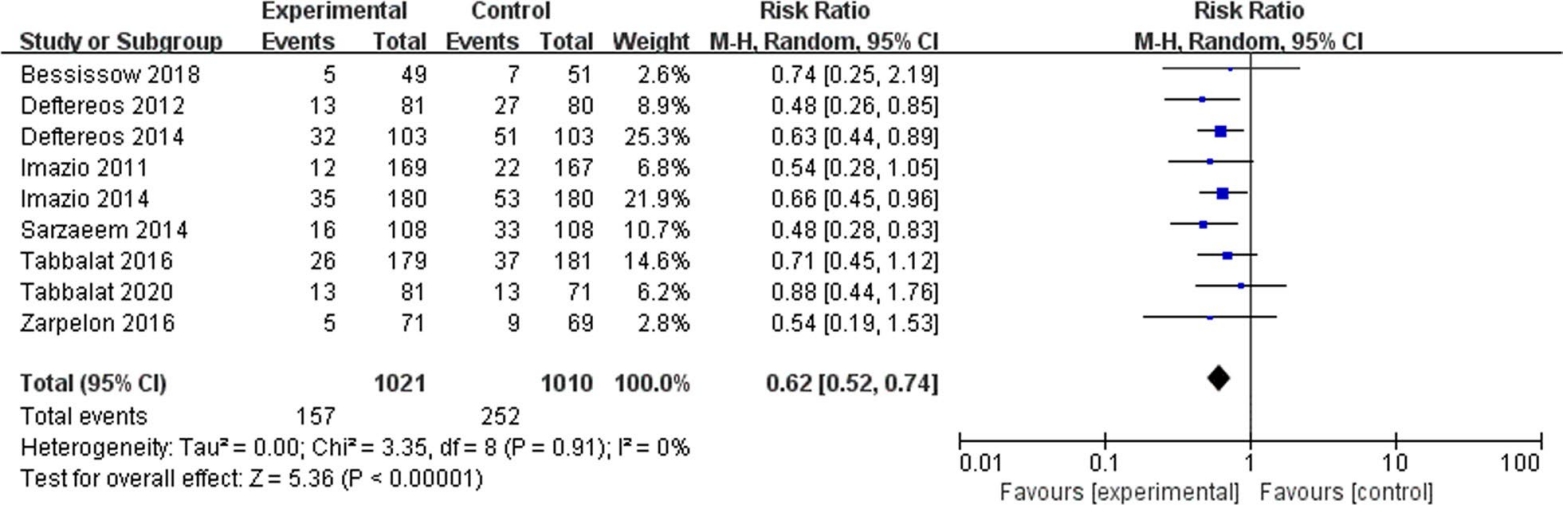
Forest plot showing estimated relative risk of POAF during colchicine use compared to placebo

**Fig. 3:**
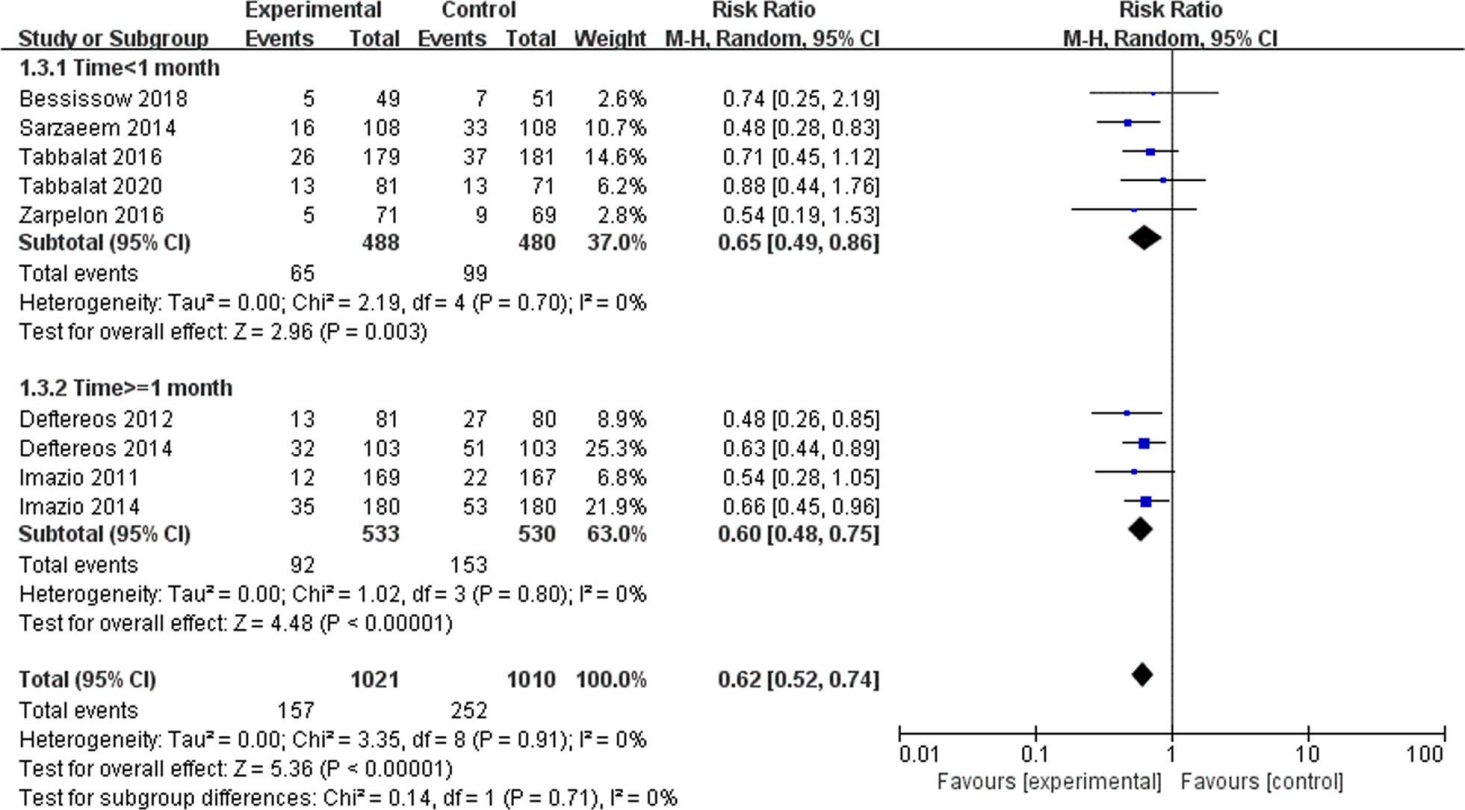
Forest plot showing estimated relative risk of POAF with different duration of colchicine use

**Fig. 4:**
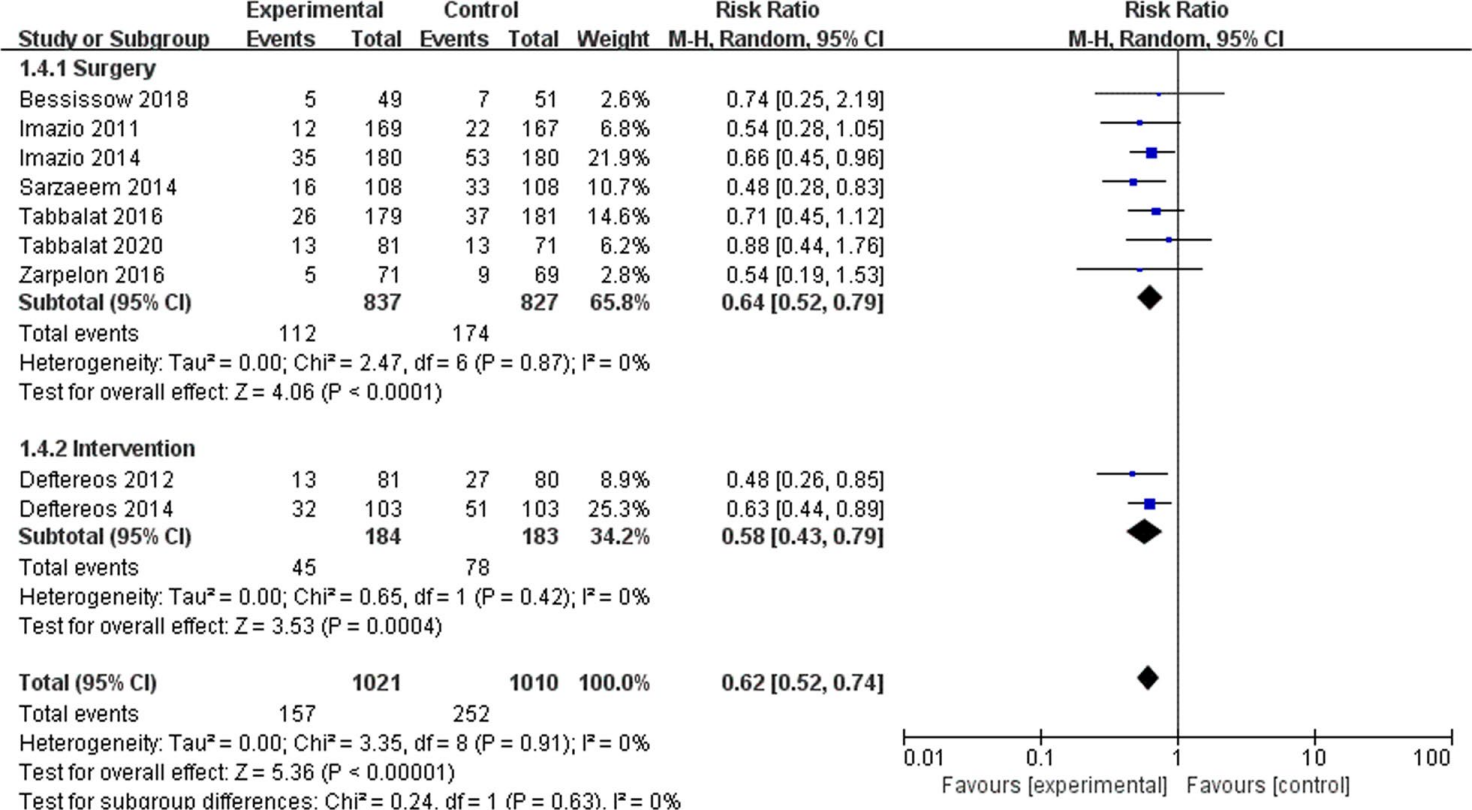
Forest plot showing estimated relative risk of POAF with respect to different surgical approach

Colchicine significantly reduced the risk of incidence of atrial fibrillation (RR 0.62; 95% CI, 0.52–0.74, *P* < 0.001, *I*^2^ = 0%) (Fig. 2). In subgroup analyses, there was a significantly lower risk of POAF for patients taking colchicine in both long-term medication group (RR 0.60; 95% CI, 0.48–0.75, *P* < 0.001, *I*^2^ = 0%), and short-term medication group (RR 0.65; 95% CI, 0.49–0.86, *P* < 0.001, *I*^2^ = 0%) compared with placebo (Fig. 3). A similar trend was observed with regard to different surgical approaches. Colchicine significantly reduced the risk of atrial fibrillation in both surgery group (RR 0.64; 95% CI, 0.52–0.79, *P* < 0.001, *I*^2^ = 0%) and intervention group (RR 0.58; 95% CI, 0.43–0.79, *P* < 0.001, *I*^2^ = 0%) compared with placebo (Fig. 4).

We observed that the risk of adverse drug reactions was significantly increased for patients taking colchicine (RR 2.65; 95% CI, 1.83–3.84, *P* < 0.001, *I*^2^ = 0%). Similar results were also found in long-term (RR 2.08; 95% CI, 1.33–3.27, *P* < 0.001, *I*^2^ = 0%) and short-term (RR 3.48; 95% CI, 2.24–5.39, *P* < 0.001, *I*^2^ = 0%) medication subgroups.

### Heterogeneity, sensitivity, bias analyses and GRADE

**Fig. 5:**
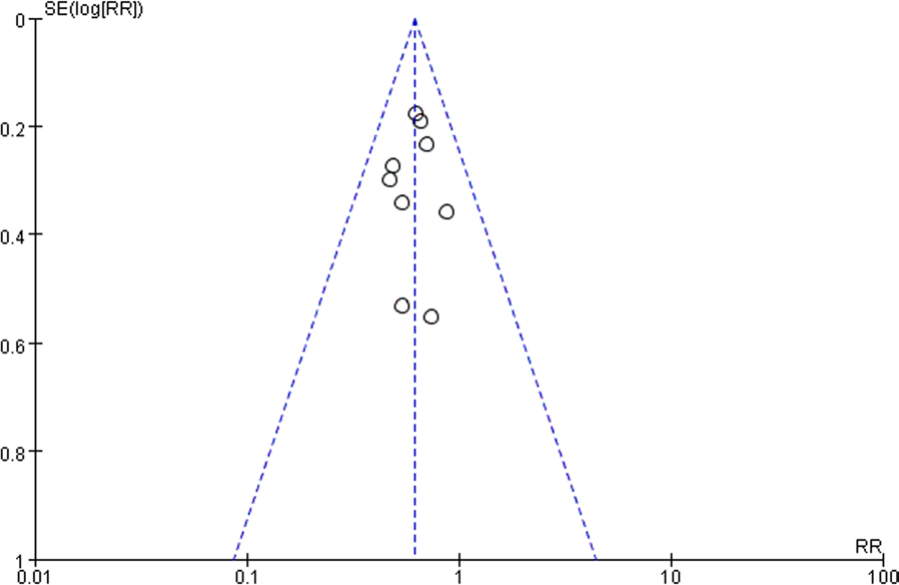
Funnel plot of standard error by estimated risk ratio

There was no evident heterogeneity (*I*^2^ < 50%) across the studies in the primary meta-analysis and subgroup analyses (Fig. 2). Sensitivity analysis suggested that the original merged result was stable. The funnel plot indicated that the likelihood of publication bias was low (Fig. 5). For the risk of bias assessment, all eight articles [15–19, 21–23] were unclear risk articles except for one article [20] which was a high-risk article. The relevant tables are placed in the Appendix. The resulting rating of GRADE is moderate, which means the study has a relatively good degree of certainty.

## Discussion

### Findings

This meta-analysis included 9 RCTs involving 2031 patients undergoing cardiothoracic surgery or cardiac intervention. To the best of our knowledge, this is the largest meta-analysis assessing the impact of colchicine on preventing POAF. Our results showed that colchicine could significantly reduce the risk of POAF, with an estimated decrease of 38%. Subgroup analyses on surgical approaches demonstrated that the risk of atrial fibrillation was significantly decreased in both surgery and intervention groups, with an estimated decrease of 36% and 42%, respectively. According to *P* = 0.63 (greater than 0.05), this indicated that the results were not statistically significantly different. The approach to surgery had little effect on the incidence of atrial fibrillation. These findings indicated that colchicine application for patients undergoing intervention and surgery might receive similar clinical benefits, and the heterogeneity caused by different surgical approaches could be ignored. Subgroup analyses on medication duration showed that the risk of atrial fibrillation was significantly reduced in both the long-term and the short-term groups, with an estimated decrease of 40% and 35%, respectively. This implied that the efficacy of colchicine in preventing atrial fibrillation might be enhanced with the increase in medication duration. However, we got *P* = 0.71 (greater than 0.05), which indicated that the effect of medication duration on the incidence of atrial fibrillation was small and not statistically significantly different. As the sample size limited our study, we hope that relevant clinical trials can be conducted in the future to investigate the effect of medication duration on the prevention of atrial fibrillation.

The adverse reactions in the test and control groups were 16.43% and 6.10%, respectively. Patients had a markedly increased risk of adverse events with the use of colchicine. The difference was significant (*P* < 0.05). We also observed that the risk of adverse reactions to colchicine in the long-term and the short-term groups increased 2.08 and 3.48 times, respectively, compared with placebo. Interestingly, the risk of adverse reactions did not increase with the extension of medication duration. Instead, the long-term subgroup had a lower incidence of adverse reactions. This may be associated with the variations in patient tolerance, sample size, and incidence of adverse reactions in each study group. A recent review of all randomized controlled trials, systematic reviews and meta-analyses of colchicine over the last two decades concluded that in the low doses used in most trials, colchicine was generally safe and well-tolerated. Gastrointestinal side effects were a notable exception, especially diarrhea in approximately 10% of colchicine users, but this was often mild, transient, and likely to resolve with dose reduction or drug discontinuation [26].

### Mechanism

The exact mechanism of POAF has not been fully elucidated [1, 27]. However, growing evidence suggests that inflammation may play an essential role during POAF development [1, 28]. Colchicine is an anti-inflammatory agent that has been used for decades in a range of acute inflammatory flares. Recent trials have demonstrated its potential in preventing the recurrence of atrial fibrillation [5]. Deftereos and colleagues measured serum inflammatory biomarkers,including C-reactive protein (CRP) and interleukin-6 (IL-6), in their two RCTs and observed that colchicine significantly decreased postoperative CRP and IL-6 levels, suggesting a reduction in inflammation levels with the use of colchicine [15, 16].

### Current treatment

In current clinical practice, rate control and rhythm control are the two major approaches for treating atrial fibrillation. Antiarrhythmic drugs (AADs), including amiodarone, metoprolol, diltiazem, are commonly used to prevent the occurrence of atrial fibrillation through rate control [29, 30]. However, the long-term effectiveness of AADs is limited, with an increased risk of ventricular arrhythmia and long-term adverse reactions [30, 31]. A previous study suggested that AADs reduced the early recurrence of atrial fibrillation after catheter ablation but could not prevent the late recurrence of arrhythmia [5]. Due to the insufficient efficacy of AADs, several non-anti-arrhythmic drugs, including anti-inflammatory drugs, have been tested in clinical trials, such as glucocorticoids, colchicine, statins, and n-3 fatty acids [5]. Non-pharmacological treatment, mainly intervention procedures, is often performed in the setting of failed medical treatment, such as radiofrequency ablation [29, 31]. However, large-scale data demonstrated that the incidence of ablation-related complications in patients with arrhythmia has also increased, which means that atrial fibrillation may recur after ablation [5, 29, 32]. Another approach is to try and restore sinus rhythm, which can be achieved by electrical or pharmacological cardioversion. Electrical cardioversion has a higher success rate than pharmacological cardioversion in the short-term [29].

Despite constant progress in treatment and expanded research efforts, the mechanism associated with the pathogenesis of atrial fibrillation, especially postoperative atrial fibrillation, remains largely unclear. Therefore, further studies are required to elucidate the underlying mechanisms of atrial fibrillation and explore optimized therapy strategies.

### Limitation

We acknowledge several limitations to this study. Given the low number of studies on low-dose colchicine, we could not analyze and compare the preventive effect of low-dose colchicine and normal-dose colchicine on POAF. This vital issue deserves further study. Moreover, the accuracy of our analysis was restricted to patient characteristics, patient compliance, the detection efficiency of atrial fibrillation, and the study design of each trial.

## Conclusion

Colchicine can effectively prevent the occurrence of atrial fibrillation after cardiothoracic surgery or cardiac intervention. The efficacy of colchicine can’t be increased over treatment duration, with no obvious adverse reactions. Future studies are needed to elucidate the optimal medication duration and dosage of colchicine to improve the treatment regimen.

## Data Availability

All data and material are available through the internet. We use revman5.0 and stata14.0 to process data. We guarantee that all data can be repeated.

## Appendix

**Table:**

Study name	Sample size	Risk of bias assessment
Bessissow [17]	100	Unclear risk
Deftereos [15]	161	Unclear risk
Deftereos [16]	206	Unclear risk
Imazio [19]	336	Unclear risk
Imazio [18]	360	Unclear risk
Sarzaeem [23]	216	Unclear risk
Tabbalat [22]	360	Unclear risk
Tabbalat [21]	152	Unclear risk
Zarpelon [20]	140	High risk
