# Correction: A meta‑analysis of colchicine in prevention of atrial fibrillation following cardiothoracic surgery or cardiac intervention

**DOI:** 10.1186/s13019-022-02030-2

**Published:** 2022-11-11

**Authors:** Hong Zhao, Yueming Chen, Min Mao, Jun Yang, Jing Chang

**Affiliations:** 1 Department of Cardiology, First Affiliated Hospital of Chongqing Medical University, Yuzhong District, Chongqing, 40000 China; 2 Department of General, Practice, The First Affiliated Hospital of Chongqing Medical University, Yuzhong District, Chongqing, 40000 China

**Correction**: Journal of Cardiothoracic Surgery (2022) 17:224 https://doi.org/10.1186/s13019-022-01958-9.

Following publication of the original article [1], the author would like to change the Result and conclusion section in abstract part

from

**Results**: A total of 9 RCTs were included in this meta-analysis, enrolling a total of 2031 patients. Colchicine significantly reduces the incidence of POAF (RR 0.62; 95% CI, 0.52–0.74, *P* < 0.001, I^2^ = 0%). Subgroup analyses indicated that the protective effect of colchicine on POAF was slightly stronger in the long-duration group (RR 0.60; 95% CI, 0.48–0.75, *P* < 0.001, I2 = 0%) than in the short-duration group (RR 0.65; 95% CI, 0.49–0.86, *P* < 0.001, I2 = 0%).

**Conclusion**: Colchicine is effective in preventing the occurrence of POAF. The efficacy of colchicine can be slightly increased over treatment duration, with no obvious adverse reactions.

to.

**Results**: A total of 9 RCTs were included in this meta-analysis, enrolling a total of 2031 patients. Colchicine significantly reduces the incidence of POAF (RR 0.62; 95% CI, 0.52–0.74, *P* < 0.001, I^2^ = 0%). Subgroup analyses indicated that the protective effect of colchicine on POAF was almost the same (*P* = 0.71) in the long-duration group (RR 0.60; 95% CI, 0.48–0.75, *P* < 0.001, I2 = 0%) and the short-duration group (RR 0.65; 95% CI, 0.49–0.86, *P* < 0.001, I2 = 0%).

**Conclusion**: Colchicine is effective in preventing the occurrence of POAF. The efficacy of colchicine cannot be increased over treatment duration, with no obvious adverse reactions.

The original article has been corrected.
